# Identification of Research Trends in Aging in Place Using Text Mining Analysis

**DOI:** 10.1093/geroni/igaa057.163

**Published:** 2020-12-16

**Authors:** Ha Neul Kim, Seok In Nam

**Affiliations:** Yonsei University, Seoul, Republic of Korea

## Abstract

Since 1980s professionals and social service providers have focused on aging at the place where people lived. This is the initial concept of the Aging in Place (AIP). Over 40 years, the topics have developed and extended to other disciplines welcoming different perspectives in the study of AIP. Therefore, this study aims to understand the overall research trends in Aging in Place (AIP) studies using text mining analysis to track the evolvement of AIP subtopics not only in Gerontology but also in various fields. To identify the topic trends, we collected the titles, abstracts, and keywords from 1,372 international articles that were published from 1981 to 2019. Then, keywords were extracted and cleaned based on precedent literature and discussions. We analyzed the keywords based on the degree of centrality and visualized the keyword-networks using VOSviewer and Pajek. Top-most popular keywords are “independent living”, “housing”, “older adults”, “home care”, “daily life activity” and “quality of life.” The change in topic trends shows that in the 1980s to early-2000s, research focused on organization and management level of intervention, home(housing) for the older adults, long term care. In the mid-2010s, health-related topics such as daily life activity, health service, health care delivery and quality of life have emerged. Recently, the topics have extended further to technology, caregiver, well-being, and environment design, environmental planning that support independent living of oneself. The research result shows that the interdisciplinary approach regarding AIP is not only inevitable but also encouraged for an in-depth discussion of the field.

